# Degree of diffuse fibrosis measured by MRI correlates with LV remodelling in childhood cancer survivors after anthracycline chemotherapy

**DOI:** 10.1186/1532-429X-13-S1-P276

**Published:** 2011-02-02

**Authors:** Edythe B Tham, Kelvin Chow, Maria Spavor, Joseph J Pagano, Mark Haykowsky, Richard Thompson

**Affiliations:** 1 University of Alberta, Edmonton, AB, Canada

## Introduction

The effectiveness of anthracycline chemotherapy is limited by dose dependant cardiotoxicity. Current protocols reduce the cumulative dose, however subclinical myocardial damage may still manifest years after cessation of therapy. The most serious side effect is dilated cardiomyopathy with pathological changes of fibrosis. The potential presence of fibrosis in this remodelling is unknown.

## Purpose

The aim of this study was to use cardiac MRI to characterize subclinical cardiac abnormalities (changes in left ventricular (LV) morphology and extent of diffuse fibrosis) in childhood cancer survivors.

## Methods

Patients with previous anthracycline therapy for childhood cancer and in remission for at least 2 years underwent cardiac MRI on a 1.5T Siemens Sonata scanner to assess LV function and mass (indexed to body surface area). Changes in myocardial T1 values from baseline to 15 minutes post bolus injection of Gd-DTPA (0.125 mmol/kg) was used to estimate the tissue concentration of contrast, reflecting diffuse fibrosis ([Gd ]= {1/T1(15 min) - 1/T1 (baseline)}/4.35, in mmol). Myocardial T1 values were quantified in 3 mid-ventricular short axis slices using a custom saturation recovery single shot SSFP pulse sequence with 10 saturation recovery times spanning the cardiac cycle, acquired in one breath-hold per slice. Myocardium was segmented using a modified AHA format with triple the circumferential resolution and mean signal intensities were fitted to a 3 parameter saturation recovery curve. Data was presented as median with ranges and analysed using Spearman’s correlation coefficient and Wilcoxon rank sum tests (p<0.05).

## Results

**Figure 1:**
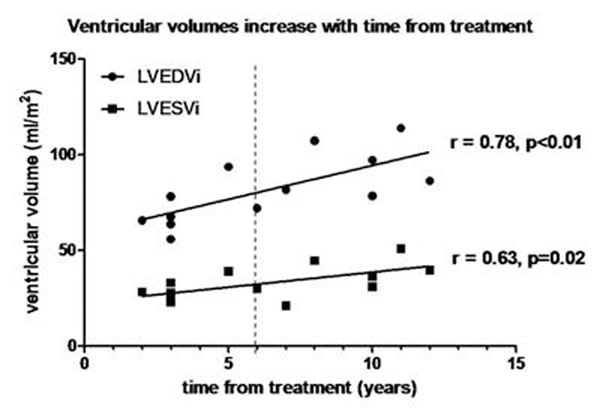


**Figure 2:**
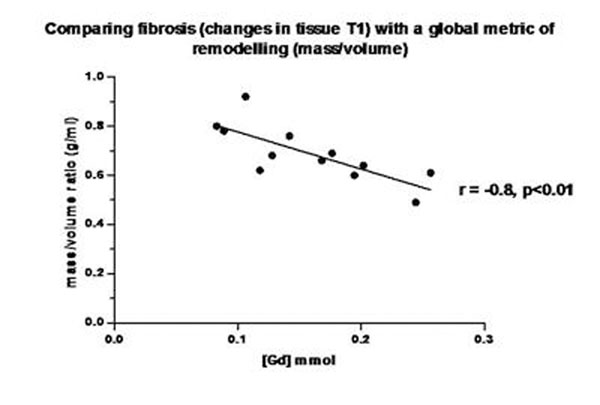


Thirteen patients (median age: 14 years, range: 9-18 years), were studied 10 years (3-12 years) after completing chemotherapy (anthracycline dose 210 mg/m^2^, range 80-375 mg/m^2^). Subjects had normal EDV 67.6 ml/m^2^ (56-114 ml/ m^2^), increased ESV 36.3 ml/m^2^ (23-60 ml/ m^2^) and normal EF (63%, range 54-74%) when compared to normal reported values in children. Ventricular volumes increased with time post chemotherapy (EDV, r=0.78, p<0.01 and ESV, r=0.63, p=0.02) (Figure 1). Furthermore, EDV and ESV increased beyond the normal range, 6-7 years post chemotherapy. Degree of fibrosis [Gd] negatively correlated with mass/volume ratio (r=-0.8, p<0.01), (Figure 2). There was no relationship between anthracycline dose and ventricular function or fibrosis.

## Conclusion

In children with previous anthracycline exposure, diffuse fibrosis as indicated by a larger myocardial concentration of Gd-DTPA was associated with ventricular dilatation without a corresponding increase in mass, (decreased mass/volume ratio). Ventricular dilatation progresses with time post therapy with larger than average values at approximately 6-7 years from chemotherapy.

